# The Impact of Recipient Demographics on Outcomes from Living Donor Kidneys: Systematic Review and Meta-Analysis †

**DOI:** 10.3390/jcm10235556

**Published:** 2021-11-26

**Authors:** Maria Irene Bellini, Mikhail Nozdrin, Liset Pengel, Simon Knight, Vassilios Papalois

**Affiliations:** 1 Department of Emergency Medicine and Surgery, Azienda Ospedaliera San Camillo Forlanini, 00152 Rome, Italy; 2 Department of Surgical Sciences, Sapienza University of Rome, 00161 Rome, Italy; 3 Imperial College School of Medicine, London SW7 2AZ, UK; mikhail.nozdrin16@imperial.ac.uk; 4 Centre for Evidence in Transplantation, Nuffield Department of Surgical Sciences, University of Oxford, Oxford OX3 7HE, UK; liset.pengel@nds.ox.ac.uk (L.P.); simon.knight@nds.ox.ac.uk (S.K.); 5 Department of Surgery and Cancer, Imperial College, London SW7 2AZ, UK; vassilios.papalois@nhs.net

**Keywords:** BMI, ethnicity, living donation, kidney transplant, recipient’s demographics

## Abstract

*Background and Aims:* Recipient demographics affect outcomes after kidney transplantation. The aim of this study was to assess, for kidneys retrieved from living donors, the effect of recipient sex, ethnicity, and body mass index (BMI) on delayed graft function (DGF) and one-year graft function, incidence of acute rejection (AR), and recipient and graft survivals. *Methods:* A systematic review and meta-analysis was performed. EMBASE and MEDLINE databases were searched using algorithms through Ovid. Web of Science collection, BIOSIS, CABI, Korean Journal database, Russian Science Citation Index, and SciELO were searched through Web of Science. Cochrane database was also searched. Risk of bias was assessed using the NHBLI tools. Data analysis was performed using Revman 5.4. Mean difference (MD) and risk ratio (RR) were used in analysis. *Results:* A total of 5129 studies were identified; 24 studies met the inclusion criteria and were analysed. Female recipients were found to have a significantly lower serum creatinine 1-year-post renal transplantation (MD: −0.24 mg/dL 95%CI: −0.18 to −0.29 *p* < 0.01) compared to male recipients. No significant difference in survival between male and female recipients nor between Caucasians and Africans was observed (*p* = 0.08). However, Caucasian recipients had a higher 1-year graft survival compared to African recipients (95% CI 0.52−0.98) with also a lower incidence of DGF (RR = 0.63 *p* < 0.01) and AR (RR = 0.55 *p* < 0.01). Recipient obesity (BMI > 30) was found to have no effect on 1-year recipient (*p* = 0.28) and graft survival (*p* = 0.93) compared to non-obese recipients although non-obese recipients had a lower rate of DGF (RR = 0.65 *p* < 0.01) and AR (RR = 0.81 *p* < 0.01) compared to obese recipients. *Conclusions:* Gender mismatch between male recipients and female donors has negative impact on graft survival. African ethnicity and obesity do not to influence recipient and graft survival but negatively affect DGF and AR rates.

## 1. Introduction

In kidney transplantation, the relative contribution of donor versus other factors on clinical outcomes is considered a main criterion to allocate an organ [1].

Living kidney donation (LKD) represents the optimal treatment for kidney failure [2,3]. Previous reports on deceased donation indicate that the donor constitution has small or moderate effect on post-transplant clinical outcomes [4], while it is widely accepted that a living donor (LD) kidney tends to function immediately, reducing the risk of hospitalisation and renal replacement therapy after transplantation to less than 4% [5] and thus setting up the recipient for the best possible result.

In the context of living donation, recipient demographics are considered equally important and are constantly evaluated as potential contraindications for an LD to come forward. For instance, there is still an ongoing debate whether or not to use a body mass index (BMI) cut-off [6], especially if that specific recipient has already one or more LDs under evaluation, in consideration of the risks related to LKD and the hypothesized inferior outcomes related to obesity [7,8].

Additionally, growing attention is being attributed to donor-recipient gender match [9] and ethnicity, in consideration of the fact that African and Asian candidates face prolonged waiting times due to difficulties in the matching process, mostly because of the scarcity of donors from these minority groups [10].

The aim of this study was to assess, for kidneys retrieved from LDs, the effect of recipient sex, ethnicity, and BMI on short- and long-term graft outcomes.

## 2. Methods

The study was registered with PROSPERO (CRD42020221109) before commencement of the literature search. The review was conducted and reported according to PRISMA guidelines [5].

### 2.1. Search Strategy

**Figure 1:**
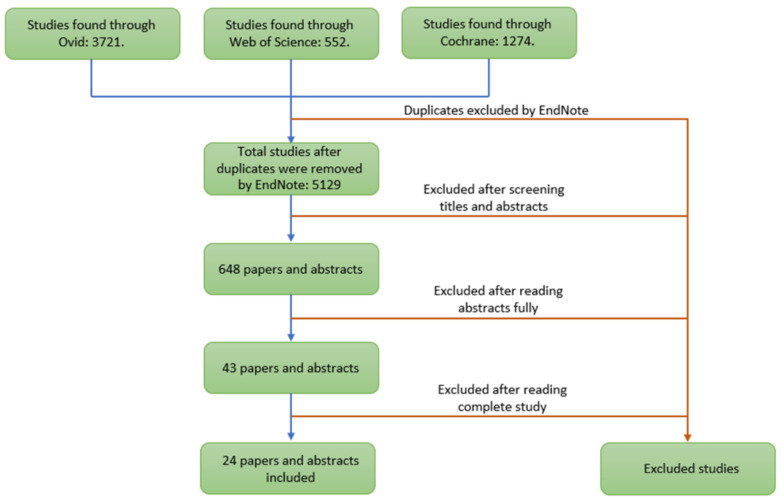
PRISMA Flowchart.

Literature searches were performed in Ovid (EMBASE, MEDLINE), Web of Science, and Cochrane databases, using combinations of free text and keyword terms for living kidney donation and donor demographics of interest. A full search strategy is shown in Appendix A (Table A1, Table A2 and Table A3). Searches were conducted on 14/11/20 and according to the PRISMA flowchart (Figure 1)

### 2.2. Inclusion/Exclusion Criteria

Any study relating to recipient’s demographic characteristics on graft outcomes after LKD were eligible for inclusion, including full articles and meeting abstracts. Only studies in English were included for the analysis.

### 2.3. Outcomes of Interest

The primary objective was to assess the effect of recipient demographics of ethnicity, BMI, and sex on kidney function evaluated using estimated glomerular filtration rate (eGFR) adjusted for body surface area, serum creatinine, and proteinuria incidence, where reported.

The secondary objectives included assessing effect of the above-mentioned recipient demographics on patient and graft survival, incidence of delayed graft function (DGF), and acute rejection (AR).

### 2.4. Screening and Data Extraction

Study identification and data extraction were performed in three stages: the first stage included downloading the studies identified by the search strategy from Cochrane, Ovid, and Web of Science databases into EndNote reference management software. The reference management software was then used to remove duplicate studies. The second stage included two independent researchers (M.I.B. and M.N.) screening the titles and abstracts of long-listed studies. The researchers then each produced a list of studies eligible for the review. The two lists were compared to produce a single short-list of studies selected for full text review. The third stage of data extraction included the researchers fully read of the short-listed studies to identify the studies meeting the inclusion criteria. Data extraction was performed by two independent reviewers (M.I.B. and M.N.), and disagreements were solved by discussion or consulting a third reviewer. Data were extracted into a Microsoft Excel sheet.

### 2.5. Risk of Bias Assessment

Risk of bias assessment was performed using National Institute of Health National Heart, Lung and Blood Institute (NIH NHBLI) quality assessment tool [6], as shown in Appendix B. Two independent reviewers, M.I.B. and M.N., judged the quality of the articles and compared their results.

### 2.6. Meta-Analysis

All data analyses were performed in Revman 5.4.1 and IBM SPSS Statistics 26. Meta-analysis of mean difference was used for continuous data. Random effect models were used for all meta-analyses due to the heterogeneous and small study samples. Mean differences with a 95% confidence interval were calculated for the summary effect. The Z test was performed to calculate *p*-values. Where *p*-values were <0.05, and 95% CI did not include 0, a statistically significant difference between the two groups was recorded. Forest plots were created in Revman 5.4.1. Heterogeneity of the data was assessed using the I2 test, where the I2 value greater than 0.5 heterogeneity of the data was assumed to be high and where the I2 value lower than 0.5 heterogeneity of the data was assumed to be low.

## 3. Results

A total of 5129 studies were identified; 24 studies met the inclusion criteria and were analysed.

### 3.1. Recipient Sex

Jacobs et al. [11] compared graft survival between male and female transplant recipients at one- and three-years post-transplantation. Wafa et al. [12] compared graft survival between male and female recipients at five- and 10- years post-transplantation. Both studies found no significant difference in short- and long-term graft survivals between male and female transplant recipients, also showing no significant difference between graft survival in transplant recipients who were the same gender as the donor and transplant recipients who were of a opposite gender as their donor. More in detail, Wafa et al. [12] found no difference between graft survival in male recipients who had received their kidney from a male or female donor, both five years and 10 years after receiving a renal transplant. The same findings were confirmed by Jacobs et al. [11], who reported no difference between graft survival in male recipients who had received their kidney from a male or female donor at one year post-transplantation; however, at three years of follow up, male recipients who had received a transplant from a male donor were 65% less likely to lose a graft compared to male recipients who received graft from a female donor (RR = 0.35; chi-square *p* = 0.006). In both studies, there was no significant difference in graft survival between females who received grafts from male and female donors.

**Figure 2:**
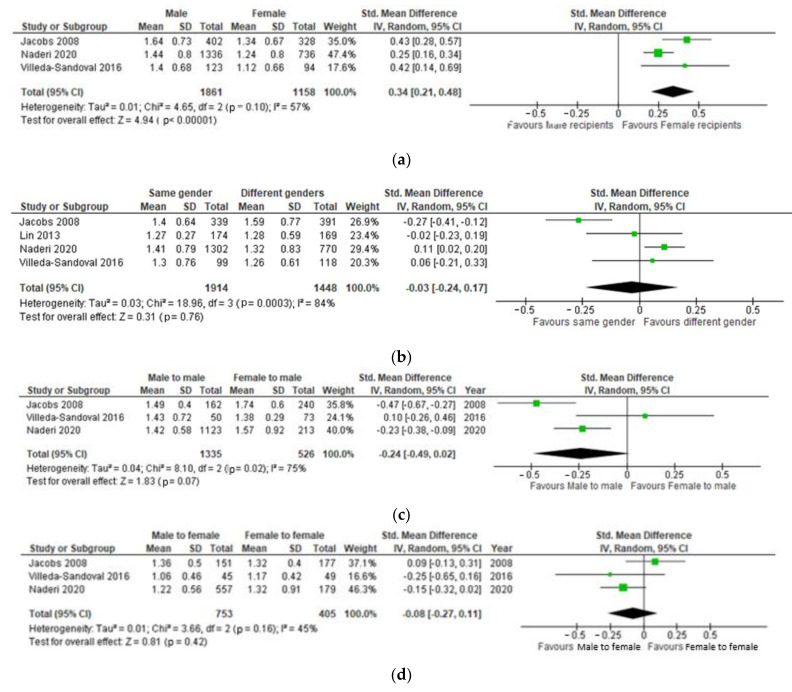

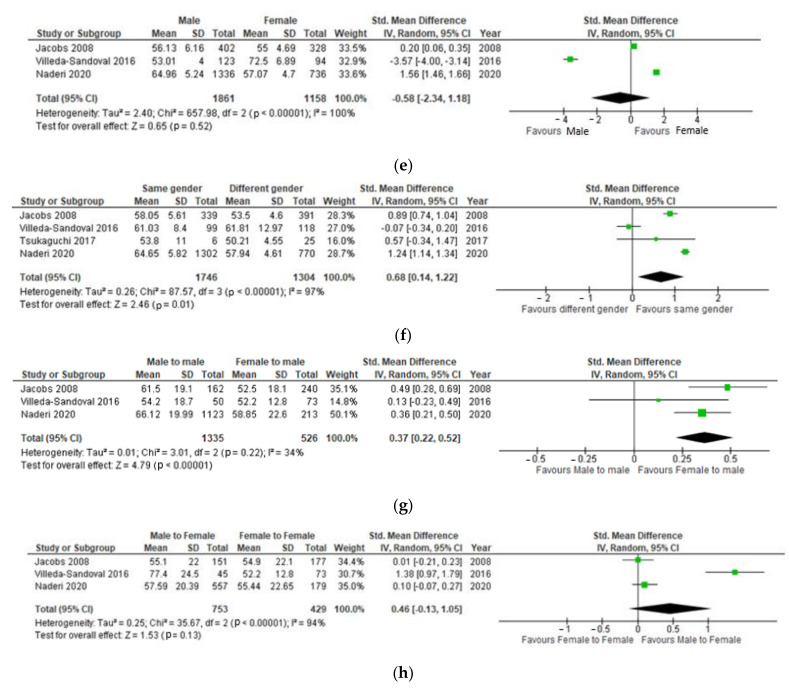
(**a**) Effect of recipient gender on serum creatinine 1-year post-transplantation. (**b**) Effect of matching genders between recipient and donor on 1-year post-transplantation serum creatinine. (**c**) One-year post-transplantation serum creatinine in male renal transplant recipients based on the gender of their donor. (**d**) One-year post-transplantation serum creatinine in female renal transplant recipients based on the gender of their donor. (**e**) One-year post-transplantation eGFR in male renal transplant recipients compared to female recipients. (**f**) Effect of matching genders of renal donor and recipient on 1-year post-transplantation eGFR. (**g**) One-year post-transplantation eGFR in male renal transplant recipients based on the gender of their donor. (**h**) One-year post-transplantation eGFR in female renal transplant recipients based on the gender of their donor.

Four studies [9,11,13,14] investigated the effect of recipient gender on the post-transplantation serum creatinine. Naderi et al. [9], Jacobs et al. [11], and Villeda-Sandoval et al. [13] compared one-year post-transplantation serum creatinine between male and female recipients of LD kidney grafts. Figure 2a shows how female recipients on average had a serum creatinine 0.24 mg/dL (0.18 to 0.29) lower than male recipients (*p* < 0.00001).

All four studies [9,11,13,14] compared one-year post-transplantation serum creatinine in recipients of kidney grafts from the same gender donors and opposite gender donors. No significant difference between recipients of renal transplants from the same gender donors and opposite gender donors (*p* = 0.78), (Figure 2b).

Three studies [8,10,12] compared one-year post-transplantation serum creatinine in male recipients receiving a transplant from male and female donors. No significant was found in one-year post-transplantation serum creatinine male recipients recovering a graft from female donors and male donors *p* = 0.06 (Figure 2c).

No significant difference in one-year post-transplantation serum creatinine was found between female recipients who had received their transplant from a male donor and female recipients who had received their transplant from a female donor (*p* = 0.22), as represented in Figure 2d.

Three studies [8,10,12] compared eGFR between male and female recipients of renal transplantation following a donation from either same gender or opposite gender donor. No significant difference in eGFR (*p* = 0.52) was found one-year post-transplantation between male and female renal transplant recipients (Figure 2e).

In Figure 2f, an important finding is that patients who received a graft from same sex donor had a significantly higher eGFR compared to recipients who received a graft from a donor of opposite sex (*p* < 0.00001). The effect size of the difference between 2 means was medium (95%CI: 0.14 to 1.22).

More in detail, male recipients who received a transplant from a male donor had a significantly higher eGFR compared to male recipients who received a transplant from a female donor (*p* < 0.00001), as represented in Figure 2g, while on the contrary, there was no significant difference in eGFR one-year post-transplantation between female recipients who received their graft from a male donor compared to those who received a graft from a female donor (*p* = 0.13) (Figure 2h).

Two studies [15,16] investigated the effect of recipient gender on the development of diabetes mellitus on grafts retrieved from LKDs. Xu et al. [15] compared the incidence of diabetes at three months of follow up, whereas Xie et al. [16] followed patients up 53.5 ± 10.4. Both studies found no significant difference between the incidence of diabetes in male and female renal transplant recipients.

Two studies [17,18] compared proteinuria between four groups: male recipients who received a transplant from a male, male recipients who received a transplant from a female, female recipients who received a transplant from male, and female recipients who received a transplant from a female. Oh et al. [18] found no significant difference in proteinuria 24 h after surgery between the four groups. On the other hand, Yanishi et al. [17] found proteinuria to be significantly lower in female recipients who had received a graft from a male donor compared to recipients who had received a transplant from the donor of the same gender as them and to male recipients who had received a renal graft from a female donor (Table 1).

### 3.2. Recipient Ethnicity

**Figure 3:**
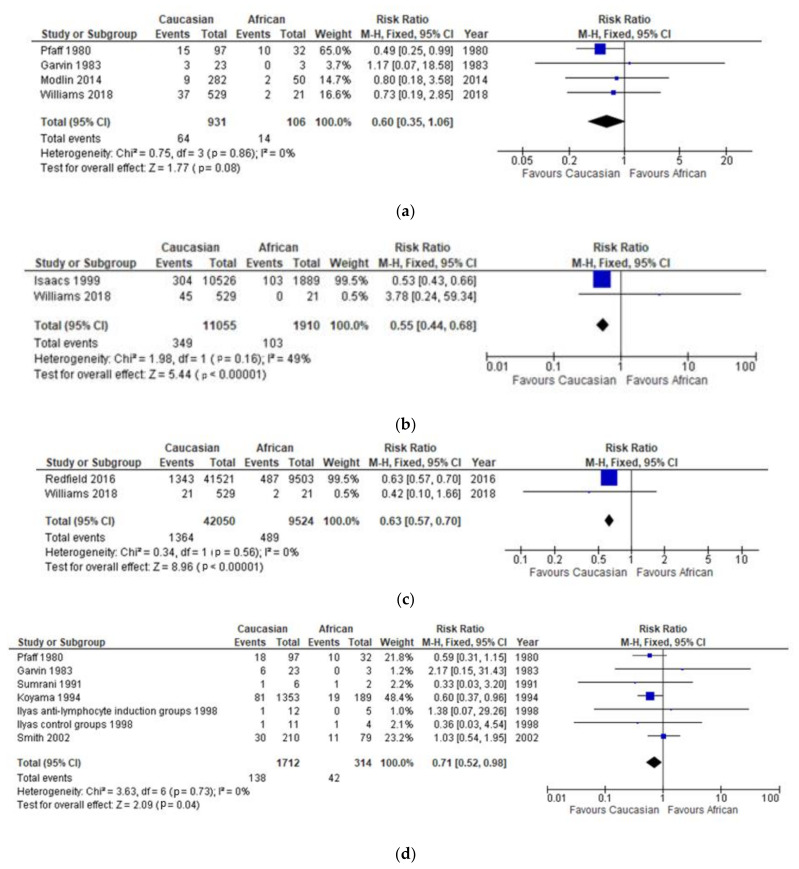
(**a**) Effect of recipient ethnicity on 1-year post-transplantation recipient survival. (**b**) Effect of recipient ethnicity on the incidence of acute rejection. (**c**) Effect of recipient ethnicity on the incidence of delayed graft function. (**d**) Effect of recipient ethnicity on 1-year graft survival.

Four studies [19,20,21,22] compared recipient survival one-year post-transplantation in Caucasian and African renal transplant recipients. There was no significant statistical difference between the recipient survival in Caucasian and African recipients (*p* = 0.88) (Figure 3a).

Williams et al. [22] and Isaacs et al. [23] compared the incidence of acute rejection in Caucasian and African recipients (Figure 3b), the latter finding a significantly lower incidence of acute rejection in Caucasian transplant recipients compared to African recipients. On the contrary, Williams et al. [11] found a higher rate of acute rejection in Caucasian recipients compared to African recipients; however, this finding was non-significant. Overall, the incidence of acute rejection post-transplantation was found to be 45% lower in Caucasian group compared to the African group; this difference was significant (*p* < 0.00001) [24].

Two studies by Williams [22] and Redfield [5] compared the incidence of DGF between Caucasian ethnicity and African ethnicity transplant recipients. Caucasian recipients were found to have a 47% lower rate of DGF following renal transplantation compared to African recipients (*p* ≤ 0.00001), as shown in Figure 3c.

Six studies compared rates of graft survival one year following renal transplantation between Caucasian and African recipients [19,20,24,25,26,27]. Ilyas et al. further split the cohorts of Caucasian and African ethnicity donors into sub-groups by whether they received anti-lymphocyte induction treatment or not. Overall, Caucasian recipients had a 29% reduced risk of losing the graft within the first year after transplantation compared to African recipients, and this difference was significant *p* = 0.04 (Figure 3d).

### 3.3. Recipient Body Mass Index

**Figure 4:**
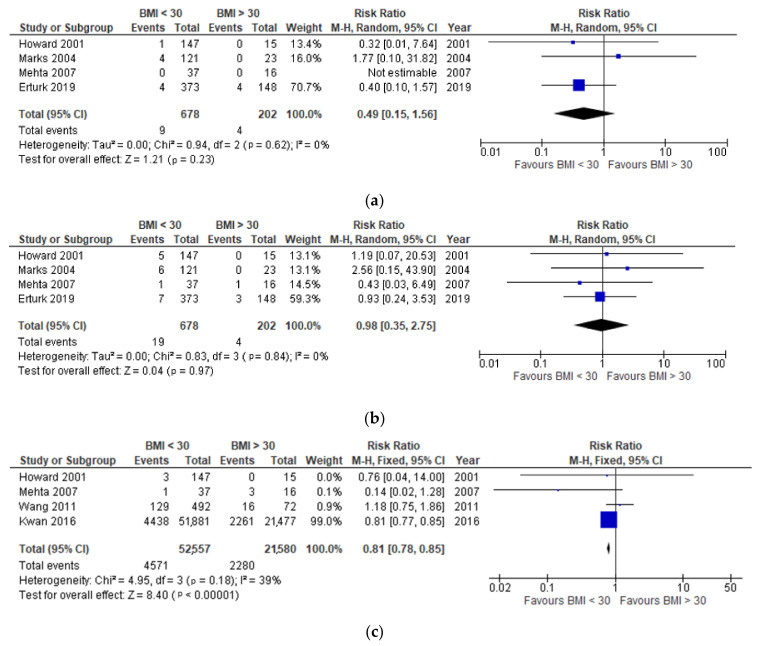

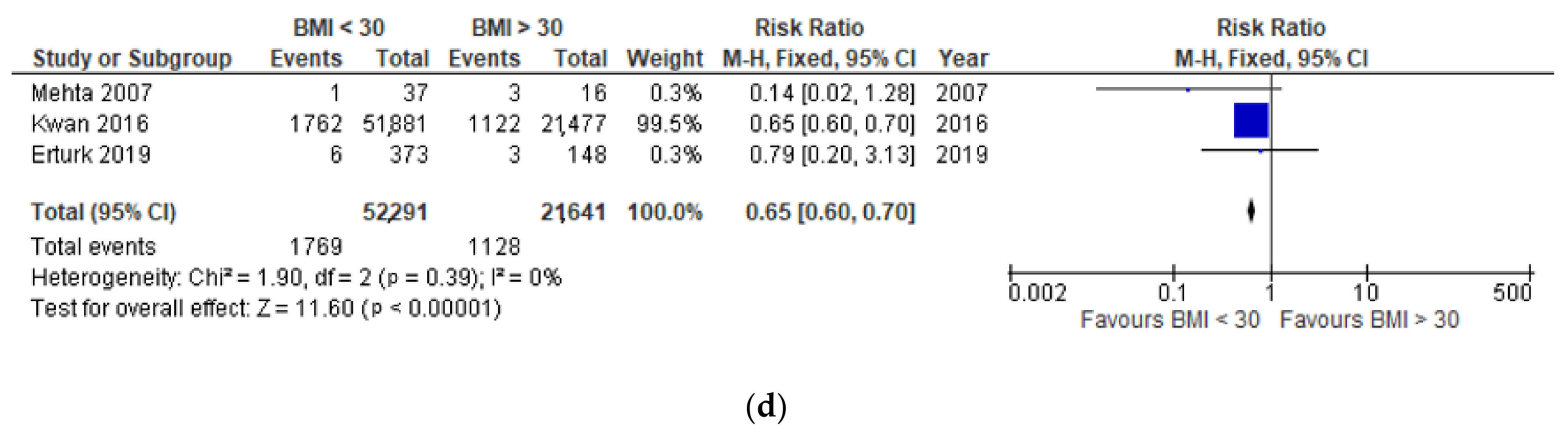
(**a**) Effect of recipient BMI on 1-year post-transplantation recipient survival. (**b**) Effect of recipient BMI on 1-year post-transplantation graft survival. (**c**) Effect of recipient BMI on the development of acute rejection. (**d**) Effect of recipient BMI on the development of delayed graft function.

Four studies investigated effect of recipient BMI on the post-transplantation one-year recipient and graft survival [28,29,30,31], finding no significant difference in obese and non-obese recipients (*p* = 0.28) (Figure 4a).

There was no significant difference between one-year graft survival in the obese and non-obese groups (*p* = 0.93), as observed in Figure 4b.

In Figure 4c, four studies [28,30,32,33] were compared to look at the difference in the acute rejection incidence between non-obese and obese recipients.

It was found that non-obese donors were 19% less likely to develop acute transplant rejection compared to obese recipients (*p* < 0.00001) (Figure 4c). Non-obese donors were also 35% less likely to develop DGF compared to obese donors (*p* < 0.00001) (Figure 4d).

## 4. Discussion

The survival advantages of transplantation over long-term dialysis are known if a given patient with end-stage kidney disease is suitable for a transplant. A major challenge is to optimize modifiable variables that could improve long-term survival [34], and with the present study, we aimed to assess the impact of recipient demographic characteristics of sex, ethnicity, and BMI on kidney grafts retrieved from LDs.

With regards to sex, an interesting finding of our meta-analysis was that at three years follow up, male recipients who had received a transplant from a male donor were 65% less likely to lose a graft compared to male recipients who have received grafts from female donors. This result might lead to think there is a nephron mass effect playing an increasing role in the medium and long-term graft function, as a female kidney could be in general of lower weight and therefore with less functional nephrons, demonstrated also by a lower eGFR in women in the general population [35]. 

In addition to this, the graft survival advantage for male recipients of male donor kidneys was previously also reported by Kayler et al. [36], who analysed the Scientific Registry of Transplant Recipients database between 1990 and 1999 and who pointed out the gender disparities in LD transplantation, with a higher proportion of wife-to-husband donations and disproportionate female-to-male donations among biological relatives and unrelated pairs.

In the present meta-analyses, we found no significant difference in graft survival among female recipients according to the sex of their donors. To this regard, as a risk factor for inferior outcomes in women, it is worth to mention the theory related to the sex-determined minor histocompatibility antigen (H-Y antigen), firstly described in 1976 on a female recipient who rejected the bone marrow transplant from her HLA-identical brother [37]. More recently, the highest number of H-Y antibodies detected in the blood of female recipients transplanted with kidneys from male donors in comparison to other sex combinations was reported to significantly correlate with the higher occurrence of acute rejection [38]. This consideration implies a careful evaluation of every possible intervention and consequent risk of sensitization in transplant patients [39]. In literature, this is supported for both deceased and living donation, as sustained by Tan et al. [40], who recommend a major focus on clinical detection of markers for minor histocompatibility loci.

Although almost significant (*p* = 0.06), the above finding was confirmed in one-year post transplant serum creatinine, with male recipients recovering better from a graft from male donors. In this view, the use of sex as a biological variable in medical research is increasingly recognized as an important modulator to better understand the complex pathophysiology of several diseases [41] and better address the future health needs. Furthermore, our study adds to the evidence that in transplantation, relevant sex-specific issues are underrecognized factors influencing patient and transplant outcomes: it is already known that women are less likely to access kidney transplantation in general, as well as transplantation from LD; therefore, whenever possible, a better gender matching is advisable for better outcomes.

This approach with a close eye to diversity and inclusion extends also to individuals from minority backgrounds: interventions to ameliorate the effects of demographic discrepancies, different ethnicity, and cultural backgrounds may improve access to transplantation [42,43] as well as transplant outcomes. From our analysis, Caucasian recipients were found to have a 47% lower rate of DGF following renal transplantation compared to African recipients (*p* = <0.00001) as well as lower AR incidence. Reasons unpinning this discrepancy are several, from different socio-economic status to prevalence of metabolic diseases [44], although a better and more inclusive allocation policy as well sensibilization of Black and Asian minorities to donate could represent an important key to improve ethnicity-related outcomes [42]; in fact, from our meta-analysis, Caucasian recipients had a 29% recused risk of losing the graft within the first year after transplantation.

Finally, the same discourse regarding discrimination could be raised with regards to high BMI recipients who are denied access to the waiting list because of their body weight only. From the present meta-analysis, the four studies investigating the effect of recipient BMI on the post-transplantation one-year recipient and graft survival [28,29,30,31] found no significant difference in obese and non-obese recipients (*p* = 0.28); therefore, even if it is true that bridge interventions, such as bariatric surgery [45], are increasingly being adopted to overcome this barrier, we think that obese patients should have the same chance as their non-obese counterparts, at least for LD renal transplantation. We also believe that obesity, as a metabolic and systemic disease, leads to higher AR and DGF rates, as per our findings; therefore, an additional effort trying to maximize all the adding risk factors to graft and patient loss is advisable, with a tailored immunosuppression [7].

## 5. Limitations

The retrospective nature of the studies analysed has limited the level of evidence we could achieve, based on observational registry data, small number of studies, and great deal of heterogeneity. Longer-term follow up reports should be also warranted to better analyse any potential relationship between the other contributing factors and the recipients’ demographics.

## 6. Conclusions

In conclusion, gender mismatch between male recipients and female donors has a negative impact on graft survival, with male recipients who received a transplant from a male donor 65% less likely to lose a graft compared to male recipients who have received grafts from female donors. African ethnicity increases DGF and AR rates compared to the Caucasian, and no significant difference between one-year graft survival in the obese and non-obese groups has been observed; therefore, BMI-only cut-offs to waitlist are not considered appropriate.

## Figures and Tables

**Table 1:** Effect of donor-recipient sex match on the graft proteinuria.

Proteinuria	Male to Male	Male to Female	Female to Female	Female to Male	Outcomes Reported in the Paper
Oh et al. Protein excretion (mg/d), 24 h urine post-op.	MM (*n* = 65): 23.4 +/− 61.6	MF (*n* = 34): 81.9 +/− 354.4	FF (=29): 9.7 +/− 51.6	FM (*n* = 67): 36.1 +/− 123.8	Independent sample *t*-test: MM-FM (*p* = 0.461), MF-FF (*p* = 0.282); MM-MF (*p* = 0.198), FM-FF: (*p* = 0.273).
Yanishi et al. (mg/day). Recipient proteinuria at 1-year post-surgery.	Group 1(same gender) *n* = 6: 135.2 ± 98.1	Group 2: (male donor to female recipient) (*n* = 8). 63.7 ± 28.7	Group 1(same gender) *n*= 6: 135.2 ± 98.1	Group 3: female donor to male recipient (*n* = 17): 205.5 ± 35.2	ANOVA between the 3 groups found the lowest proteinuria to be in the Male to Female group (*p* < 0.01).

## Data Availability

The data used to support the findings of this study are included within the article.

## Abbreviations

AR, acute rejection; BMI, body mass index; DGF, delayed graft function; eGFR, estimated glomerular filtration rate; LD, living donor; LKD, living kidney donation.

## Appendix A. Search Strategy

EMBASE and MEDLINE databases were searched through Ovid on 14/11/2020, the search algorithm used is shown in Table A1. English language filter was applied to the search.

**Table A1:** Search algorithm used to search EMBASE and MEDLINE databases through Ovid.

Step	Input
1	gender/ or "gender and sex"/
2	sex/ or sex difference/
3	sex
4	age/
5	ethnicit*
6	ethnic minorit*
7	BAME
8	exp "ethnic or racial aspects"/
9	BMI/
10	BMI or weight
11	genetic relationship/
12	1 or 2 or 3 or 4
13	5 or 6 or 7 or 8 or 9 or 10 or 11
14	12 and 13
15	exp kidney donor/
16	kidney transplantation/
17	living donor/
18	exp graft recipient/
19	15 or 16 or 17 or 18
20	14 and 19

Web of Science core collection, BIOSIS (1950-2008), CABI, Korean Journal database, Russian Science Citation Index and SciELO were searched through Web of Science search engine on 14/11/2020. The search algorithm used is shown in Table A2.

**Table A2:** Search algorithm used to search Web of Science core collection, BIOSIS (1950-2008), CABI, Korean Journal database, Russian Science Citation Index and SciELO through Web of Science.

Step	Input
1	TS=(sex or gender)
2	TS=(sex and difference)
3	TS=age
4	TS=(ethnicit* or ethnic minorit*)
5	TS=BAME
6	TS=(ethnic* or race)
7	TS=(BMI or weight)
8	TS=genetic relationship
9	#1 or #2 or #3
10	#4 or #5 or #6 or #7 or #8
11	#9 and #10
12	TS=kidney
13	TS=transplantation
14	TS=(living or live or non-deceased)
15	TS=(donor)
16	TS=graft
17	TS=recipient
18	#12 and #13 and #14 and #15 and #16 and #17
19	#11 and #18

Cochrane library database was searched on 14/11/2020. The search algorithm used is shown in Table A3.

**Table A3:** Search algorithm used to search the Cochrane library database.

Step	Input
1	MeSH descriptor: [Gender Identity] this term only
2	MeSH descriptor: [Sex] this term only
3	MeSH descriptor: [Sex Characteristics] this term only
4	(sex):ti,ab,kw
5	MeSH descriptor: [Age Factors] this term only
6	ethnicit*
7	ethnic minorit*
8	BAME
9	BMI
10	weight
11	MeSH descriptor: [Family] explode all trees
12	genetic relationship
13	MeSH descriptor: [Ethnic Groups] explode all trees
14	MeSH descriptor: [Continental Population Groups] explode all trees
15	#1 or #2 or #3 or #4 or #5
16	#6 or #7 or #8 or #9 or #10 or #11 or #12 or #13 or #14
17	#15 or #16
18	MeSH descriptor: [Kidney] explode all trees
19	MeSH descriptor: [Tissue Donors] explode all trees
20	MeSH descriptor: [Transplantation] explode all trees
21	MeSH descriptor: [Transplant Recipients] explode all trees
22	#18 and #19
23	#18 and #20
24	#18 and #21
25	#22 or #23 or #24
26	Kidney 51158
27	donor or transplantation or recipient or transplant
28	#26 and #27
29	#17 AND #25
30	#17 AND #28
31	#29 or #30

## Appendix B. Risk of Bias Assessment

**Reference number**	**Country of study**	**Authors**	**1. Was the resarch question clearly stated?**	**2. Was the study population clearly specified and defined?**	**3. Was the participation of eligible persons at least 50%?**	**4. Were inclusion and exclusion criteria for being in the study prespecified and applied uniformly to all participants?**	**5. Was a sample size justification, power description, or variance and effect estimates provided?**	**6. For the analyses in this paper, were the exposure(s) of interest measured prior to the outcome(s) being measured?**	**7. Was the timeframe sufficient so that one could reasonably expect to see an association between exposure and outcome if it existed?**	**8. For exposures that can vary in amount or level, did the study examine different levels of the exposure as related to the outcome (e.g., categories of exposure, or exposure measured as continuous variable)?**	**9. Were the exposure measures (independent variables) clearly defined, valid, reliable, and implemented consistently across all study participants?**	**10. Was the exposure(s) assessed more than once over time?**	**11. Were the outcome measures (dependent variables) clearly defined, valid, reliable, and implemented consistently across all study participants?**	**12. Were the outcome assessors blinded to the exposure status of participants?**	**13. Was loss to follow-up after baseline 20% or less?**	**14. Were key potential confounding variables measured and adjusted statistically for their impact on the relationship between exposure(s) and outcome(s)?**	**Quality Rating (Good, Fair or Poor)**
[5]	USA	Redfield, R.R., et al.	Yes	Yes	Yes	Yes	Yes	Yes	Yes	No	Yes	Not applicable	Yes	No	Yes	Yes	Good
[9]	Iran	Naderi, G., et al.	Yes	Yes	Yes	Yes	No	Yes	Yes	Not applicable	Yes	Not applicable	Yes	No	Yes	No	Fair
[11]	USA	Jacobs, S.C., et al.	Yes	Yes	Yes	Yes	No	Yes	Yes	Not applicable	Yes	Not applicable	Yes	No	Yes	Yes	Good
[12]	Egypt	Wafa, E.W., et al.	Yes	Yes	Yes	Yes	No	Yes	Yes	Not applicable	Yes	Not applicable	Yes	No	Yes	No	Fair
[13]	Mexico	Villeda-Sandoval, C.I., et al.	Yes	Yes	Yes	Yes	No	Yes	Yes	Not applicable	Yes	Not applicable	Yes	No	Yes	No	Fair
[14]	China	Lin, J., et al.	Yes	Yes	Yes	Yes	No	Yes	Yes	Not applicable	Yes	Not applicable	Yes	No	Yes	No	Fair
[15]	China	Xu, J., et al.	Yes	Yes	Yes	Yes	No	Yes	Yes	Not applicable	Yes	Not applicable	Yes	No	Yes	No	Fair
[16]	China	Xie, L., et al.	Yes	Yes	Yes	Yes	No	Yes	Yes	Not applicable	Yes	Not applicable	Yes	No	Yes	No	Fair
[17]	Japan	Yanishi, M., et al.	Yes	Yes	Yes	Yes	No	Yes	Yes	Not applicable	Yes	Not applicable	Yes	No	Yes	No	Fair
[18]	South Korea	Oh, C.-K., et al.	Yes	Yes	Yes	Yes	No	Yes	Yes	Not applicable	Yes	Not applicable	Yes	No	Yes	No	Fair
[19]	USA	Pfaff, W.W., et al.	No	Yes	Yes	Yes	No	Yes	Yes	No	Yes	Not applicable	Yes	No	Yes	No	Fair
[20]	USA	Garvin, P.J., et al.	Yes	Yes	Yes	Yes	No	Yes	Yes	No	Yes	Not applicable	Yes	No	Yes	No	Fair
[21]	USA	Modlin, C.S., et al.	YEs	Yes	Yes	Yes	No	Yes	Yes	No	Yes	Not applicable	Yes	No	Yes	Yes	Good
[22]	UK	Williams, A., et al.	Yes	Yes	Yes	Yes	Yes	Yes	Yes	No	Yes	Not applicable	Yes	No	Yes	Yes	Good
[23]	USA	Isaacs, R.B., et al.	Yes	Yes	Yes	Yes	Yes	Yes	Yes	No	Yes	Not applicable	Yes	No	Yes	yes	Good
[24]	USA	Ilyas, M., et al.	Yes	Yes	Yes	Yes	No	Yes	Yes	No	Yes	Not applicable	Yes	No	Yes	No	Fair
[26]	USA	Koyama, H. et al.	Yes	Yes	Yes	Yes	Yes	Yes	Yes	No	Yes	Not applicable	Yes	No	Yes	Yes	Good
[27]	USA	Smith, S.R. et al.	Yes	Yes	Yes	Yes	No	Yes	Yes	No	Yes	Not applicable	Yes	No	Yes	Yes	Good
[28]	USA	Howard, R.J., et al.	YEs	Yes	No	Yes	No	Yes	Yes	No	Yes	Not applicable	Yes	No	Not stated	No	Fair
[29]	USA	Marks, W.H., et al.	Yes	Yes	Yes	Yes	No	Yes	Yes	No	Yes	Not applicable	Yes	No	Yes	No	Fair
[30]	USA	Mehta, R., et al.	Yes	Yes	YEs	Yes	No	Yes	Yes	No	Yes	Not applicable	Yes	No	Yes	No	Fair
[31]	Turkey	Erturk, T. et al.	Yes	Yes	Yes	Yes	No	Yes	Yes	No	Yes	Not applicable	Yes	No	Not stated	No	Fair
[32]	China	Wang, K. et al.	No	Yes	Yes	Yes	No	Yes	Yes	No	Yes	Not applicable	Yes	No	Yes		
[33]	USA	Kwan, J.M., et al.	Yes	Yes	Yes	Yes	Yes	Yes	Yes	No	Yes	Not applicable	Yes	No	Yes	Yes	Good

## References

[1] Policies and guidance ODT. https://www.odt.nhs.uk/transplantation/tools-policies-and-guidance/policies-and-guidance/.

[2] LaPointe R.D., Hays R., Baliga P., Cohen D.J., Cooper M., Danovitch G.M., Dew M.A., Gordon E.J., Mandelbrot D.A., McGuire S. (2015). Consensus conference on best practices in live kidney donation: Recommendations to optimize education, access, and care. American journal of transplantation. Am. J. Transplant..

[3] Bellini M.I., Courtney A.E., McCaughan J.A. (2020). Living Donor Kidney Transplantation Improves Graft and Recipient Survival in Patients with Multiple Kidney Transplants. J. Clin. Med..

[4] Kerr K.F., Morenz E.R., Thiessen-Philbrook H., Coca S.G., Wilson F.P., Reese P.P., Parikh C.R. (2019). Quantifying Donor Effects on Transplant Outcomes Using Kidney Pairs from Deceased Donors. Clin. J. Am. Soc. Nephrol..

[5] Redfield R.R., Scalea J.R., Zens T.J., Muth B., Kaufman D.B., Djamali A., Astor B.C., Mohamed M. (2016). Predictors and outcomes of delayed graft function after living-donor kidney transplantation. Transpl. Int..

[6] Bellini M.I., Paoletti F., Herbert P.E. (2019). Obesity and bariatric intervention in patients with chronic renal disease. J. Int. Med. Res..

[7] Bellini M.I., Koutroutsos K., Galliford J., Herbert P.E. (2017). One-Year Outcomes of a Cohort of Renal Transplant Patients Related to BMI in a Steroid-Sparing Regimen. Transpl. Direct.

[8] Bellini M.I., Koutroutsos K., Nananpragasam H., Deurloo E., Galliford J., Herbert P.E. (2020). Obesity affects graft function but not graft loss in kidney transplant recipients. J. Int. Med. Res..

[9] Naderi G., Azadfar A., Yahyazadeh S.R., Khatami F., Aghamir S.M.K. (2020). Impact of the donor-recipient gender matching on the graft survival from live donors. BMC Nephrol..

[10] Purnell T.S., Luo X., Cooper L.A., Massie A.B., Kucirka L.M., Henderson M.L., Gordon E.J., Crews D.C., Boulware E., Segev D.L. (2018). Association of Race and Ethnicity With Live Donor Kidney Transplantation in the United States From 1995 to 2014. Jama.

[11] Jacobs S.C., Nogueira J.M., Phelan M.W., Bartlett S.T., Cooper M. (2008). Transplant recipient renal function is donor renal mass- and recipient gender-dependent. Transpl. Int..

[12] Wafa E.W., Shokeir A., Akl A., Hassan N., Fouda M.A., El Dahshan K., Ghoneim M.A. (2011). Effect of donor and recipient variables on the long-term live-donor renal allograft survival in children. Arab. J. Urol..

[13] Villeda-Sandoval C.I., Rodríguez-Covarrubias F., Martinez A.G.-C.Y., Lara-Nuñez D., Guinto-Nishimura G.Y., González-Sánchez B., Magaña-Rodríguez J.D., Alberú-Gómez J., Vilatobá-Chapa M., Gabilondo-Pliego B. (2016). The impact of donor-to-recipient gender match and mismatch on the renal function of living donor renal graft recipients. Gac. Med. Mex..

[14] Lin J., Zheng X., Xie Z.-L., Sun W., Zhang L., Tian Y., Guo Y.-W. (2013). Factors potentially affecting the function of kidney grafts. Chin. Med. J..

[15] Xu J., Xu L., Wei X., Li X., Cai M. (2018). Incidence and Risk Factors of Posttransplantation Diabetes Mellitus in Living Donor Kidney Transplantation: A Single-Center Retrospective Study in China. Transplant. Proc..

[16] Xie L., Tang W., Wang X., Wang L., Lu Y., Lin T. (2016). Pretransplantation Risk Factors Associated With New-onset Diabetes After Living-donor Kidney Transplantation. Transplant. Proc..

[17] Yanishi M., Tsukaguchi H., Huan N.T., Koito Y., Taniguchi H., Yoshida K., Mishima T., Sugi M., Kinoshita H., Matsuda T. (2017). Correlation of whole kidney hypertrophy with glomerular over-filtration in live, gender-mismatched renal transplant allografts. Nephrology.

[18] Oh C.-K., Lee B.M., Jeon K.O., Kim H.J., Pelletier S.J., Kim S.I., Kim Y.S. (2006). Gender-related differences of renal mass supply and metabolic demand after living donor kidney transplantation. Clin. Transplant..

[19] Pfaff W.W., Morehead R.A., Fennell R.S., Mars D.R., Thomas J.M., Brient B.W. (1980). The role of various risk factors in living related donor renal transplant success. Ann. Surg..

[20] Garvin P.J., Castaneda M., Codd J.E., Mauller K. (1983). Recipient race as a risk factor in renal transplantation. Arch. Surg..

[21] Modlin C.S., Alster J.M., Saad I.R., Tiong H.Y., Mastroianni B., Savas K.M., Zaramo C.E., Kerr H.L., Goldfarb D., Flechner S.M. (2014). Renal Transplantations in African Americans: A Single-center Experience of Outcomes and Innovations to Improve Access and Results. Urology.

[22] Williams A., Richardson C., McCready J., Anderson B., Khalil K., Tahir S., Nath J., Sharif A. (2018). Black Ethnicity is Not a Risk Factor for Mortality or Graft Loss After Kidney Transplant in the United Kingdom. Exp. Clin. Transpl..

[23] Isaacs R.B., Nock S.L., Spencer C.E., Connors A.F., Wang X.-Q., Sawyer R., Lobo P.I. (1999). Racial disparities in renal transplant outcomes. Am. J. Kidney Dis..

[24] Ilyas M., Ammons J.D., Gaber A.O., Iii. S.R., Batisky D.L., Chesney R.W., Jones D.P., Wyatt R. (1998). Comparable renal graft survival in African-American and Caucasian recipients. Pediatric Nephrol..

[25] Sumrani N., Delaney V., Hong J.H., Daskalakis P., Markell M., Friedman E.A., Sommer B.G. (1991). Racial differences in renal transplant outcome of insulin-dependent diabetic recipients in the cyclosporine era. ASAIO Trans..

[26] Koyama H., Cecka J.M., Terasaki P.I. (1994). Kidney transplants in black recipients. HLA matching and other factors affecting long-term graft survival. Transplantation.

[27] Smith S.R., Butterly D.W. (2002). Declining influence of race on the outcome of living-donor renal transplantation. Am. J. Transpl..

[28] Howard R.J., Thai V.B., Patton P.R., Hemming A.W., Reed A., Van Der Werf W.J., Fujita S., Karlix J.L., Scornik J.C. (2002). Obesity does not portend a bad outcome for kidney transplant recipients. Transplantation.

[29] Marks W.H., Florence L.S., Chapman P.H., Precht A.F., Perkinson D.T. (2004). Morbid obesity is not a contraindication to kidney transplantation. Am. J. Surg..

[30] Mehta R., Shah G., Leggat J., Hubbell C., Roman A., Kittur D., Narsipur S. (2007). Impact of recipient obesity on living donor kidney transplant outcomes: A single-center experience. Transpl. Proc..

[31] Erturk T., Berber I., Cakir U. (2019). Effect of Obesity on Clinical Outcomes of Kidney Transplant Patients. Transpl. Proc..

[32] Wang K., Liu Q.Z. (2011). Effect Analysis of 1-Year Posttransplant Body Mass Index on Chronic Allograft Nephropathy in Renal Recipients. Transplant. Proc..

[33] Kwan J.M., Hajjiri Z., Metwally A., Finn P.W., Perkins D.L. (2016). Effect of the Obesity Epidemic on Kidney Transplantation: Obesity Is Independent of Diabetes as a Risk Factor for Adverse Renal Transplant Outcomes. PLoS ONE.

[34] Hariharan S., Israni A.K., Danovitch G. (2021). Long-Term Survival after Kidney Transplantation. N. Engl. J. Med..

[35] Piccoli G.B., Alrukhaimi M., Liu Z.-H., Zakharova E., Levin A. (2018). What We Do and Do Not Know about Women and Kidney Diseases; Questions Unanswered and Answers Unquestioned: Reflection on World Kidney Day and International Women’s Day. Nephron.

[36] Kayler L.K., Rasmussen C.S., Dykstra D.M., Ojo A.O., Port F.K., Wolfe R.A., Merion R.M. (2003). Gender imbalance and outcomes in living donor renal transplantation in the United States. Am. J. Transpl..

[37] Tan J.C., Wadia P.P., Coram M., Grumet F.C., Kambham N., Miller K., Pereira S., Vayntrub T., Miklos D.B. (2008). H-Y antibody development associates with acute rejection in female patients with male kidney transplants. Transplantation.

[38] Graňák K., Kováčiková L., Skálová P., Vnučák M., Miklušica J., Laca ., Mokáň M., Dedinská I. (2020). Kidney Transplantation and "Sex Mismatch": A 10-Year Single-Center Analysis. Ann. Transpl..

[39] Bellini M.I., Charalmpidis S., Brookes P., Hill P., Dor F.J.M.F., Papalois V. (2019). Bilateral Nephrectomy for Adult Polycystic Kidney Disease Does Not Affect the Graft Function of Transplant Patients and Does Not Result in Sensitisation. Biomed Res. Int..

[40] Tan J.C., Kim J.P., Chertow G.M., Grumet F.C. (2012). Donor–Recipient Sex Mismatch in Kidney Transplantation. Gend. Med..

[41] Bairey Merz C.N., Dember L.M., Ingelfinger J.R., Vinson A., Neugarten J., Sandberg K.L., Sullivan J.C., Maric-Bilkan C., Rankin T.L., on behalf of the participants of the National Institute of Diabetes and Digestive and Kidney Diseases Workshop on “Sex and the Kidneys” (2019). Sex and the kidneys: Current understanding and research opportunities. Nat. Rev. Nephrol..

[42] Taylor D.M., Bradley J.A., Bradley C., Draper H., Dudley C., Fogarty D., Fraser S.D., Johnson R., Leydon G.M., Metcalfe W. (2019). Limited health literacy is associated with reduced access to kidney transplantation. Kidney Int..

[43] Bellini M.I., Charalampidis S., Stratigos I., Dor F., Papalois V. (2019). The Effect of Donors’ Demographic Characteristics in Renal Function Post-Living Kidney Donation. Analysis of a UK Single Centre Cohort. J. Clin. Med..

[44] Pruthi R., Robb M.L., Oniscu G.C., Tomson C., Bradley A., Forsythe J.L., Metcalfe W., Bradley C., Dudley C., Johnson R.J. (2020). Inequity in Access to Transplantation in the United Kingdom. Clin. J. Am. Soc. Nephrol..

[45] Ku E., McCulloch C.E., Roll G.R., Posselt A., Grimes B.A., Johansen K.L. (2021). Bariatric surgery prior to transplantation and risk of early hospital re-admission, graft failure, or death following kidney transplantation. Am. J. Transpl..

